# Unraveling the Relationship between Trait Negative Affectivity and Habitual Symptom Reporting

**DOI:** 10.1371/journal.pone.0115748

**Published:** 2015-01-20

**Authors:** Katleen Bogaerts, Liselotte Rayen, Ann Lavrysen, Ilse Van Diest, Thomas Janssens, Koen Schruers, Omer Van den Bergh

**Affiliations:** 1 Health Psychology, University of Leuven, Leuven, Belgium; 2 Movement Control and Neuroplasticity Research Group, University of Leuven, Leuven, Belgium; 3 School for Mental Health and Neuroscience, Maastricht University, Maastricht, The Netherlands; Queen Mary University of London, UNITED KINGDOM

## Abstract

**Objective:**

In two studies, we aimed at further elucidating the relationship between trait negative affectivity (NA) and habitual symptom reporting (HSR) by relating these variables to measures of executive function, trait questionnaires, and effects of emotion induction.

**Methods:**

Healthy female participants (N = 75) were selected on their scores for trait NA and for the Checklist for Symptoms in Daily Life. Three groups were compared: (1) low NA-low HSR; (2) high NA-low HSR; and (3) high NA-high HSR (low NA-high HSR did not occur). In study 1, participants underwent a Parametric Go/No-go Task and a Stroop Color-Word test, and trait questionnaires measured alexithymia and absorption. Forty-five participants (N = 15 in each group) were further engaged in study 2 to induce state NA using an affective picture paradigm.

**Results:**

Impaired inhibition on the Stroop and Go/No go Task characterized high trait NA, but not high HSR, whereas alexithymia and absorption were elevated in HSR, regardless of trait NA. Negative picture viewing induced elevated state NA in all groups, but only high HSR also reported more bodily symptoms. This effect was moderated, but not mediated by state NA.

**Conclusion:**

High trait NA is a vulnerability factor but not a sufficient condition to develop HSR. Deficient inhibition is related to the broad trait of NA, whereas the moderating effect of state NA on symptom reporting is specific for high HSR. Understanding processes related to alexithymia and absorption may specifically help to explain elevated HSR.

## Introduction

Medically unexplained symptoms (MUS)—symptoms not related to a known physiologic dysfunction—form a heterogeneous group of complaints involving a variety of bodily systems [1]. The share of MUS in primary care is estimated to range from 20% up to 50%, while prevalence rates in secondary care are even higher [2–3]. Also in the non-clinical population there is a large group of persons experiencing MUS in daily life (a.k.a. habitual symptom reporters, HSR, [4]).

A personality trait that consistently correlates with symptom reports is negative affectivity (NA), the tendency to experience negative affect or mood [5–6], which is generally more elevated in women compared to men [7]. A correlation of .40 to .50 between NA ratings and symptom reports is typically found in clinical as well as non-clinical populations [8], while NA-related differences in objective health status are rarely found [9–11] suggesting that high NA is associated with reporting more unfounded (i.e., independent of objective markers of disease) symptoms [10]. Also in well-defined diseases, NA may be related to elevated symptom reports but this shows up mainly when the symptoms of a condition are ambiguous (e.g. diabetes) or overlap with, and/or are difficult to differentiate from psychophysiological responses associated with emotional distress [12]. Also other studies showed that trait NA is more associated with vague, systemic complaints than with specific, localized symptoms [8], possibly due to the larger semantic overlap between emotional and physical distress in case of vague symptoms [13]. However, despite the widely reported and robust positive correlation between trait NA and habitual symptom reporting, research that explores the mechanisms underlying this relationship is scarce.

The present studies intended to investigate this relationship in further depth from a symptom perception perspective. In line with recent theorizing [1, 14], it is assumed that the experience of symptoms relies on an automatic integration of information from the body (bottom-up) with perceptual-cognitive schemata in memory (top-down; i.e., memory representations of a personal learning history with somatic experiences—including related beliefs, expectations, and negative affective connotations). MUS arise due to chronic activation of schemata, and minimal or no peripheral physiological changes are necessary to activate the symptom experience. The brain apparently interprets these activated schemata as being actual symptom episodes.

Symptom schemata can easily be activated by associated cues. Experiments of our group creating MUS in the laboratory by use of repeated inhalations of odorous air enriched with CO_2_, have established that the mere presence of harmless odor cues associated with previous symptom episodes can trigger elevated symptom reports. However, such “learned MUS” were more easily established [1] in psychosomatic patients (compared with healthy controls) and in healthy people reporting high negative affect and/or high levels of symptoms in daily life, and [2] when the predictive cue was (made) unpleasant [15–17]. Bogaerts et al. [18] further showed that the within-subject correspondence between respiratory self-reported symptoms and experimentally induced changes in the respiratory physiology is reduced in high trait NA persons, in high HSR persons and in a clinical MUS population, especially in a symptom-related or a distressing context [19, 20].

In addition, studies applying emotional stimulation during somatic experiences show more interference between emotional and somatic information in MUS. Montoya et al. [21] found that when somatic stimulation took place within an aversive emotional context, abnormal processing of somatosensory information occurred in fibromyalgia patients, compared to patients with pain resulting from identifiable somatic lesions. In a study by Bogaerts et al. [22], emotional states were induced by simply viewing sets of negative, neutral, positive or symptom-related pictures (International Affective Picture System (IAPS); [23]) without concomitant somatic stimulation. All participants reported higher levels of state NA during the negative and symptom-related picture series compared with the positive and neutral picture series, but only high HSR also reported more bodily symptoms after viewing the negative and symptom-related pictures. These results pointed to a learned association between negative emotional states and symptom reporting in high habitual symptom reporters that is activated by negative picture viewing.

Because every person has schematic representations of symptom episodes in memory associated with emotional states but not MUS, it is claimed that the main problem in MUS is a lack of inhibition of the schemata [1] and/or a deficient filtering of low intensity noisy information from the peripheral body [14]. In either case, it can reasonably be assumed that a deficient executive control function is related to the experience of MUS.

The idea of a deficient inhibition system in MUS is compatible with brain imaging findings in patients with irritable bowel syndrome [24], fibromyalgia [25], and temporomandibular disorder [26]. Fibromyalgia patients performed worse on the Parametric Go/No-go Task [27], compared with healthy controls, as well as on the Stroop Color-Word task compared with patients with memory complaints who did not have fibromyalgia [28]. Both cognitive tasks are indirect measures of prefrontal inhibitory control of pre-potent responses [29–31], suggesting difficulties with response inhibition for people with high trait NA as well as for those suffering from MUS in daily life.

In the present studies, we investigated the relationship between trait negative affectivity (NA) and habitual symptom reporting (HSR), using emotion induction and indirect measures of prefrontal inhibitory control. For practical reasons only women were recruited. However, because MUS are more prevalent in women than in men, the study is relevant for a majority of MUS patients [2]. In a first study, we hypothesized that persons with low trait NA would show better prefrontal inhibition than those with high trait NA. Because high trait NA appears as a necessary but not sufficient condition to develop HSR, an interesting research question was whether HSR-status would add to this effect. For exploratory reasons, we further included two trait questionnaires measuring variables (alexithymia, absorption) often related to trait NA and/or MUS in literature. A body of evidence (see [32] for an overview) suggests that individuals with alexithymia are significantly more likely to exhibit unexplained medical phenomena than those who do not display this tendency [33]. The inability to experience and express one’s own feelings has been suggested to lead to the misinterpretation of emotion-induced physiological activity as somatic symptoms [34–35]. Absorption—or hypnotisability—may confer vulnerability for MUS through its relation with self-focused attention [36].

In a second experimental study, part of the same sample was invited to participate in a study using the affective picture paradigm [22]. We wanted to replicate our earlier findings in an fMRI-compatible design, and extend the observation that inductions of brief state NA can trigger MUS reporting in a nonmedical, neutral context (which was not controlled in Bogaerts et al, [22]). We also wanted to exploratorily investigate the relationship between the data collected in study 1 and the amount of symptoms triggered in study 2.

## Method

### Participants

Healthy female students (N = 75, 18–26 years) participated in return for course credits or 20 euros. They were selected on their scores on the Dutch trait version of the Positive and Negative Affect Schedule (PANAS-trait; [7]) and the Checklist for Symptoms in Daily Life (CSD; [37]). Cut-off scores (based on 394 female students, scale range 39–139; SD = 16.15, see also [19–20]; [22]) were used to obtain extreme groups of low (score < 21) *versus* high (score > 29) trait NA persons and low (score < 75) *versus* high (score > 100) habitual symptom reporters. Only those participants meeting the CSD and PANAS inclusion criteria on both a collective screening some weeks before and at the moment of participation in the study were included in the study. Since high HSR did not co-occur with low NA in the population, three groups could eventually be compared: (1) low NA-low HSR (*n* = 30); (2) high NA-low HSR (*n* = 15); and (3) high NA-high HSR (*n* = 30).

Exclusion criteria were medical conditions, such as cardiovascular, gastrointestinal, neuromuscular, pulmonary, acute illnesses or psychiatric conditions—other than somatisation disorder or undifferentiated somatoform disorder [1], and pregnancy. The experimental protocol, including the informed consent form, was considered in accordance with the Declaration of Helsinki [38] of the World Medical Association and was approved by the Multidisciplinary Ethical Committee of the Department of Psychology and by the Medical Ethics Committee of the University of Leuven, Belgium. All participants provided written informed consent.

### Stimuli

**Stroop Color-Word Task.**The Stroop Color-Word Task [30] consists of three subtasks. The first subtask shows color words on a white cardboard card in random order printed in black ink (Card A). Subtask 2 displays solid color patches in one of these four basic colors (Card B). Subtask 3 contains color words printed in an incongruous ink color (Card C). The participants were instructed to read the words, name the colors, and finally, name the ink color of the printed words as quickly and accurately as possible in the three subsequent subtasks. The Stroop interference effect is calculated by subtracting the time needed to complete card C minus the time needed to complete card B. The number of errors on Card C is also indicative for inhibition capacity. The Stroop Color-Word Test is considered a reliable assessment tool [39].

**Parametric Go/No-go Task.**The Parametric Go/No-go Task [31] is a computer task, consisting of three subtasks, in which the participants have to react on the specific letters x, y, and z by pressing on a target key as fast as possible. During the task several other distracting letters are shown as well. The first task (Level 1) is a sustained attention or vigilance task requiring to respond to the targets x, y, and z whenever they appear. On level 2 contextual inhibition is required: the participant has only two targets (x and y) and must shift between the to-be-responded and the to-be-inhibited targets based upon the last target to which they responded. Once an x has been responded to, the participant may only respond to a letter y. If they responded to the y, they may only react upon the letter x and so on. Level 3 increases the difficulty and requires set-shifting between three targets. The participant receives targets, x, y, and z, and must shift cognitive set between the to-be-responded and the to-be-inhibited targets based upon the last target to which they responded before. Convergent validity and test-retest reliability of this task have been demonstrated [31].

**Affective picture paradigm.**The affective picture paradigm is similar to the one used in Bogaerts et al. [22] with the exception that timing parameters of stimulus presentation and symptom reporting were changed to make it fMRI compatible. Also, it did not use symptom-related pictures since the latter study did not found any difference in results between symptom-related pictures and general negative affective ones. We selected 216 photographs from the International Affective Picture System (IAPS; Center for the Study of Emotion and Attention (CSEA-NIMH; [23]) categorized into neutral, positive and negative pictures (72 pictures per category). Pictures were equated using norms for valence and arousal [23] and negative pictures had similar fear, sadness and disgust ratings, using normative data collected by Mikels et al. [40]. Only low disgust pictures were included in the sample [40]. More detailed information about picture content can be found in the IAPS manual [23] and on the following website: http://csea.phhp.ufl.edu/media/iapsmessage.html. The selection can be found in S1 Text

**Figure 1:**
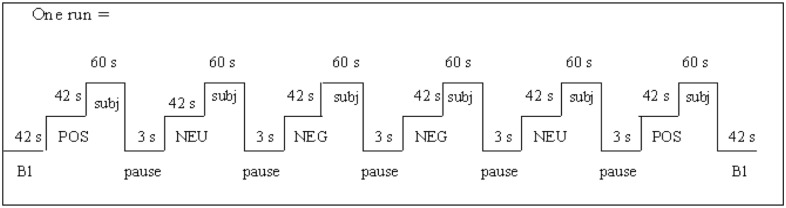
Schematic representation of one run consisting of six blocks of pictures. (Bl: baseline; POS: positive pictures (6 pictures presented for 7s each per block); NEU: neutral pictures; NEG: negative pictures; subj: subjective ratings).

Six pictures per emotion block were presented on a 19-inch flat screen monitor in a randomized order (7s per picture). Six runs of six consecutive blocks (two blocks of each emotion per run requiring 12 different pictures per emotion category; runs and blocks were counterbalanced, see Fig. 1 depicting one run), with an intermittent presentation of ten symptoms from a symptom checklist (Tightness of the chest; Pounding of the heart; Stomach/abdominal cramps; Headache; Fatigue; Not being able to breathe deeply enough; Rapid heartbeat; Nausea; Dizziness; Muscular pain) and ten negative affective adjectives from the PANAS-state (Distressed; Nervous; Hostile; Guilty; Scared; Upset; Jittery; Irritable; Ashamed; Afraid). Participants indicated after each picture series on a five-point scale to what extent they experienced the symptoms and emotions during the picture series (1 = not at all, 2 = a little bit, 3 = moderate, 4 = rather strongly, 5 = very strongly). Pictures were presented using AFFECT 4.0 software [41].

### Measures

**Trait questionnaires.**The Checklist for Symptoms in Daily Life (CSD) contains the original 35 items from Wientjes and Grossman [37] and four additional items (see [37, 42] for components and reliability). This questionnaire assesses habitual symptom reporting on a five-point scale (never, seldom, sometimes, often, very often).

The trait version of the Positive Affect and Negative Affect Schedule (PANAS; [7, 43]) consists of ten positive (Positive Affectivity; PA) and ten negative (Negative Affectivity; NA) adjectives. Participants rate their feelings in general (very slightly or not at all, a little, moderately, quite a bit, extremely).

The Toronto Alexithymia Scale (TAS-20) consists of 20 statements which have to be rated on a five-point scale (ranging from strongly disagree to strongly agree). Subscales are (1) Difficulty identifying one’s feelings and distinguishing between emotions and bodily sensations; (2) Difficulty in describing one’s feelings; (3) Tendency towards externally-oriented thinking. Internal consistency, test-retest reliability, and validity have been confirmed [35].

The Tellegen Absorption Scale aims to measure “openness to absorbing and self-altering experiences” [44] and is related to hypnotisability [45]. It consists of 34 statements and participants are asked to indicate whether these sentences apply to them in general (true or false). Internal and test-retest reliability have been documented [46].

### Procedure

Participants were invited to engage in two studies. In study 1 they signed an informed consent form and were asked about their medical status, using a set of specific health questions to ensure that the symptoms in daily life reported by the high habitual symptom reporters could not be attributed to a medical condition. They also completed the CSD and the PANAS-trait on a computer. Subsequently, a manual version of the Stroop Task [30] and a computerized Parametric Go/No-go Task [31] were completed. The participants also filled out the paper-and-pencil version of the TAS-20 and the Tellegen Absorption Scale.

Forty-five participants (15 in each group) of the original sample were invited to participate in a second study another time. They signed the informed consent, were seated in a dimly lit room and received a non-connected headphone to dim surrounding sounds. A training trial let participants become acquainted with the rating scales. Between each of the six runs (see Fig. 1), there was a one minute pause, during which the lights were turned on. Finally, the participants were given a manipulation check asking for the purpose of the studies.

### Statistical analysis

**Study 1.** We performed two ANOVAs (Statistica) on the results of the Stroop Task and the Parametric Go/No-go Task, with Group (2 levels) as a between-subject variable. First, we compared the high NA group (N = 45, collapsing high NA-low HSR and high NA-high HSR) with the low NA group (low NA-low HSR; N = 30). In a second series of analyses, we compared high with low HSR within the high NA group (group high NA—high HSR; N = 30; group high NA—low HSR; N = 15). Dependent variables were reaction time (RT) and number of errors for the Stroop Task, and % correct No-go for the Parametric Go/No-go Task. ANOVAs with Group (3 levels: low NA-low HSR, high NA-low HSR, high NA-high HSR) were performed on the (sub)scores of the Toronto Alexithymia Scale and the Tellegen Absorption Scale. Data are provided in S2 File.

**Study 2.** State NA and symptom reporting were investigated using repeated measures ANOVAs with Picture type (3 levels: positive, neutral, negative) and Run (6 levels) as within-subject variables and Group (3 levels: low NA-low HSR, high NA-low HSR, high NA-high HSR) as a between-subject variable. Multilevel mediation/moderation analysis (using R, [47–48]) was performed to examine the role of state NA as a mediator, respectively, moderator of the association between HSR and symptom reporting.

Outliers were removed whenever necessary using boxplots and Cook’s distance. Greenhouse–Geisser corrections were applied when appropriate, *ε* values and corrected *p* values are reported. Follow-up comparisons were made with (Bonferroni-corrected) Tukey HSD a posterioritests. Non-parametric tests were used when data were not normally distributed. Trend analyses were performed whenever proper. The *α* for all analyses was set at 0.05. Effect sizes are indicated by *η_p_^2^.* Data are provided in Data S1, S2 and S3 in S1 File (see Supporting Information Legend).

## Results

### Study 1: Prefrontal inhibition and questionnaires

**Figure 2:**
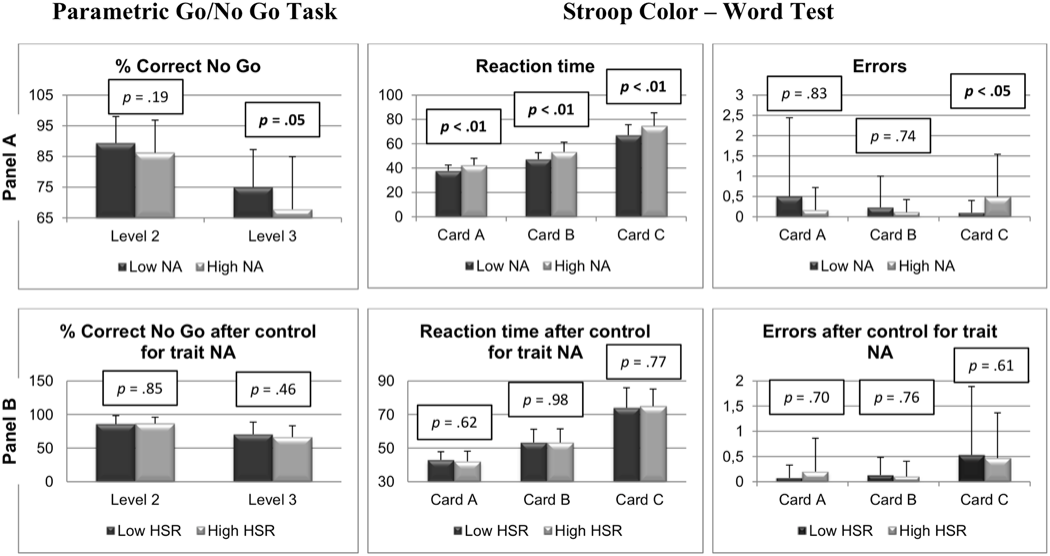
Percentage of correct No-Go responses during the second and third subtask of the Parametric Go/No-go Task before (left panel A) and after (left panel B) control for trait NA. Reaction time to the different cards of the Stroop Task before (middle panel A) and after (middle panel B) control for trait NA. Number of errors on the different cards of the Stroop Task before (right panel A) and after (right panel B) control for trait NA. Whiskers denote standard errors of means.

**Parametric Go/No Go Task.**On Level 3, low trait NA persons had a higher percentage of correct No Go’s than high trait NA persons, *F*(1,73) = 3.97, *p* = .05, *η_p_^2^* = .05 (Fig. 2, left panel A), whereas these groups did not differ on Level 2, *F*(1,70) = 1.77, *p* = .19, *η_p_^2^* = .02. Within the high trait NA group, low and high HSR persons did not differ on any of the levels (Level 2: *F*(1,40) = .03, *p* = .85, *η_p_^2^* = .00, Level 3: *F*(1,43) = .55, *p* = .46, *η_p_^2^* = .01 (Fig. 2, left panel B).

**Stroop Color-Word Test.**Low trait NA persons showed faster reaction times than high trait NA persons for all cards (A: *F*(1,72) = 13.99, *p* < .01, *η_p_^2^* = .16, card B: *F*(1,73) = 13.67, *p* < .01, *η_p_^2^* = .16, card C: *F*(1,73) = 10.72, *p* < .01, *η_p_^2^* = .13 (Fig. 2, middle panel A). Within the high trait NA group, low and high HSR persons did not differ on any of the cards (card A: *F*(1,42) = .25, *p* = .62, *η_p_^2^* = .01, Card B: *F*(1,43) = .00, *p* = .98, *η_p_^2^* = .00, Card C: *F*(1,43) = .09, *p* = .77, *η_p_^2^* = .00, Fig. 2, middle panel B). No differences between groups emerged as regards the Stroop interference effect (RT Card C minus RT Card B).

Low trait NA persons made significantly less errors compared with high trait NA persons, yet only on Card C (Card A, *U* = 664.5, *p* = .83; Card B, *U* = 657.5, *p* = .74; Card C, *U* = 518.5, *p* < .05; Fig. 2, right panel A). Within high trait NA, errors did not differ between low and high HSR persons (Card A, *U* = 216.5, *p* = .70; Card B, *U* = 217.5, *p* = .76; Card C, *U* = 208, *p* = .61; Fig. 2, right panel B).

**Figure 3:**
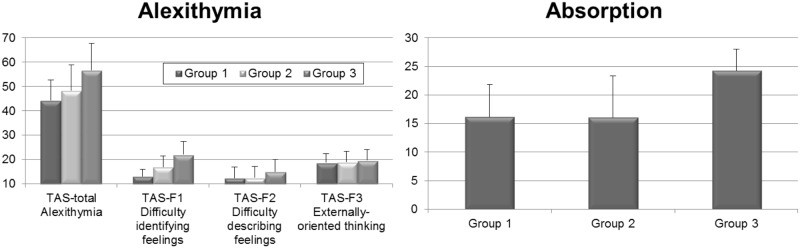
Scores on the Toronto Alexithymia Scale (TAS-total) with its three subscales (TAS-F1, TAS-F2, TAS-F3; left panel) and scores on the Tellegen Absorption Scale (right panel) for group 1 (low NA-low HSR), group 2 (high NA-low HSR), and group 3 (high NA-high HSR). Whiskers denote standard errors of means.

**Questionnaires.** Fig. 3 (left panel) shows that the groups differed for alexithymia, *F*(2,72) = 11.89, *p* < .01, *η_p_^2^* = .25. Tukey HSD tests highlighted the importance of HSR-status: The high NA-high HSR group had significantly higher total scores compared with the other two groups which did not differ. A similar picture was seen for subscale TAS-F2 (“Difficulty describing feelings”). For subscale TAS-F1 (“Difficulty identifying feelings”) scores of all groups differed from each other, whereas for subscale TAS-F3 (“Externally-oriented thinking”) no differences between the groups appeared (See Table 1 for *p* values).

**Table 1:** Bonferroni-tests and *p* values for alexithymia (and three subscales) and absorption for the three groups.

***Group***	**TAS-total**	**TAS-F1**	**TAS-F2**	**TAS-F3**	**Absorption**
*1 vs. 2*	*p* = .21	*p* < .01	*p* = .96	*p* = .92	*p* = .95
*1 vs. 3*	*p* < .01	*p* < .01	*p* < .05	*p* = .44	*p* < .01
*2 vs. 3*	*p* < .01	*p* < .01	*p* = .09	*p* = .60	*p* < .01

*Notes:* TAS-total = Total score on Toronto Alexithymia Scale; TAS-F1 = Difficulty Identifying Feelings; TAS-F2 = Difficulty Describing Feelings; TAS-F3 = Externally-Oriented Thinking

HSR emerged also as an important variable for the scores of the Tellegen Absorption Scale: High NA-high HSR had higher scores than the other two groups which did not differ [Group: *F*(2,72) = 20.18, *p* < .01, *η_p_^2^* = .36; see Table 1 for *p* values).

### Study 2: Emotion induction by means of the affective picture paradigm

**Figure 4:**
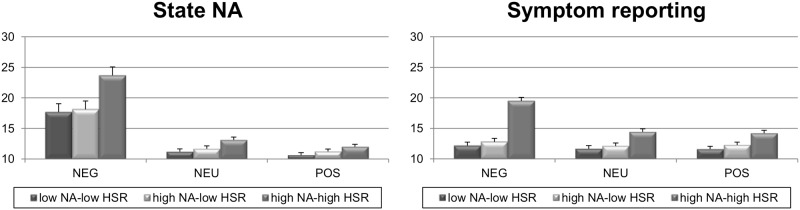
Changes in state NA (left panel) and symptom score (right panel) over the different picture series [negative (NEG); neutral (NEU); positive (POS)] for low NA-low HSR persons, high NA-low HSR persons, and high NA-high HSR persons. Whiskers denote standard errors of means. The minimum score on these questionnaires is 10.

**State NA.** Fig. 4, left panel, shows that state NA was overall higher during negative picture viewing compared with neutral (*p* < .0001) and positive picture viewing (*p* < .0001), whereas the latter two had no different effects (Picture Type: *F*(2,84) = 105.23, *p* < .0001, *ε* = .53, *η_p_^2^* = .71). Also, the high NA-high HSR group reported overall higher state NA than the low NA-low HSR group (*p* < .01) and the high NA-low HSR group (*p* < .01), whereas the latter two groups did not differ (Group: *F*(2,42) = 8.41, *p* < .001, *η_p_^2^* = .29).

However, during negative picture viewing high NA-high HSR persons reported significantly more state NA than low NA-low HSR persons (*p* < .001) and high NA-low HSR persons (*p* < .001) whereas the latter two groups did not differ (Group x Picture type interaction, *F(*4,84) = 3.42, *p* < .05, *ε* = .53, *η_p_^2^* = .14). During neutral and positive picture viewing, no significant group differences were found. None of the effects involving Run were significant.

**Symptoms.**High NA-high HSR reported overall more symptoms than high NA-low HSR (*p* < .00001) and the low NA-low HSR (*p* < .00001) [Group: *F(*2,42) = 24.47, *p* < .0001, *η_p_^2^* = .54; the latter two groups did not differ]. Also negative picture viewing induced overall more symptoms compared with neutral (*p* < .00001) and positive picture viewing (*p* < .00001) [Picture type: *F*(2,84)=51.11, *p* < .0001, *ε* = .61, *η_p_^2^* = .55; the latter two types did not differ]. However, Fig. 4 (right panel) shows that during negative picture viewing, high NA-high HSR persons reported substantially more symptoms than the other two groups (*p* < .00001), which did not differ (Group x Picture type: *F*(4,84) = 27.46, *p* < .0001, *ε* = .61, *η_p_^2^* = .57). High NA-high HSR persons reported also more symptoms than low NA-low HSR persons (*p* < .05) during neutral and positive picture viewing, whereas the high NA-low HSR persons did not differ from both other groups. Importantly, high NA-high HSR persons reported significantly more symptoms during negative picture viewing compared with neutral and positive picture viewing (*p* < .00001), while showing no difference between the latter two, whereas symptom reporting in the low NA-low HSR and the high NA-low HSR groups was not influenced by the different picture series.

All persons reported more symptoms towards the end of the experiment [Run: *F*(5,210 = 6.13, *p* < .01, *ε* = .39, *η_p_^2^* = .13; linear trend, *p* < .01]. This did not interact with Group, *F*(10,210) = .98, *p* = .42, *ε* = .39, *η_p_^2^* = .04, but it did with Picture type. Symptom reports linearly increased for the neutral (*p* < .001), and for positive pictures (*p* < .001), but not for the negative pictures (*p* = .36) [Run x Picture type: *F*(10,420) = 2.79, *p* < .05, *ε* = .57, *η_p_^2^* = .06].

**Multilevel mediation/moderation analysis.**Four conditions had to be fulfilled for state NA being a mediator in the relationship between HSR and symptom reporting. Firstly, multilevel analysis (model 1) showed that HSR could predict symptom reporting, *F*(2,43) = 24.47, *p* < .0001. Secondly, HSR was a significant predictor of state NA (model 2; *F*(2,43) = 8.41, *p* < .001). Thirdly, model 3 showed that symptom reporting could be significantly predicted by state NA, *F*(1,43)=47.95, *p* < .0001. A fourth condition, the effect of HSR as a predictor of symptom reporting should become non-significant after adding state NA to the model, was not met: when state NA was added to the model, the effect of HSR remained significant, *F*(2,43) = 17.77, *p* < .0001. Therefore, we can conclude that state NA is—at best—only a partial mediator. Moderation effects were tested by including the interaction between HSR and state NA in the model (model 4), showing a significant effect, *F*(2,43) = 4.14, *p* < 0.05, which indicated that state NA is a moderator in the relationship between HSR and symptom reporting (Table 2).

**Table 2:** Multilevel mediation/moderation analysis.

	**Model 1: IV—> DV**			**Model 2: IV—> M (state NA)**			**Model 3: IV, M—> DV**			**Model 4: including HSR x state NA interaction**		
*Fixed effects*	F	df	p	F	df	p	F	df	p	F	df	p
HSR	24.47	2;43	<.0001	8.41	2;43	0.0008	17.77	2;43	<.0001	0.10	2;43	0.9041
Picture Type	29.21	2;43	<.0001	55.33	2;43	<.0001	0.78	2;43	0.4667	0.96	2;43	0.3925
HSR x Picture Type	16.43	4;43	<.0001	1.79	4;43	0.1481	14.86	4;43	<.0001	1.54	4;43	0.2072
State NA							47.95	1;43	<.0001	43.90	1;43	<.0001
HSR x State NA										4.14	2;43	0.0227
*Random effects estimates (covariance structure: unstructured)*												
cov (neg, neg)	180.45			1026.34			165.31			182.10		
cov (neu, neg)	89.9095			121.09			95.6479			111.58		
cov (neu, neu)	135.79			112.18			107.68			112.41		
cov (pos, neg)	90.3127			-5.0873			96.7616			111.67		
cov (pos, neu)	121.49			72.9794			93.9669			98.7556		
cov (pos, pos)	125.57			86.4127			97.9463			102.49		
*Fit statistics Deviance*	906.2			1010.0			876.6			876.1		
AIC	918.2			1022.0			888.6			888.1		
BIC	929.1			1032.9			899.5			898.9		

### Explorative analyses

As a further exploration, we wanted to check whether inadequate inhibitory capacity, alexithymia, or absorption were related to the elevated symptom reporting in high HSR. In order to do so, we calculated Pearson’s correlations between the symptom induction effect (the difference in symptom reports between negative and neutral picture viewing in study 2) and variables that have shown significant between group differences (study 1: % correct no go level 3; Stroop RT card C; number of mistakes card C; Alexithymia total; Alexithymia F1; Alexithymia F2; Absorption total score). A significant positive correlation between the symptom difference score and F1 (difficulty identifying feelings; *r* = .43; *p* < .01) and absorption total score (*r* = .44; *p* < .01) emerged. Adding the latter variables as covariates to the ANOVA’s on the symptom reports in study 2 did not alter the main effect of Group nor the Group x Picture interaction. However, adding absorption did undo the main effect of Picture type, *F*(2,82) = 2.91, *p* = .06, *ε* = .61, *η_p_^2^* = .07, whereas the latter remained significant after adding Alexithymia F1, *F*(2,82) = 6.30, *p* < .01, *ε* = .61, *η_p_^2^* = .13. Adding interaction effects (Absorption x Picture type, F1 x Picture type) did not alter the effects.

## Discussion

The present studies aimed to investigate the intriguing link between trait NA and HSR. Three groups differing on trait NA and HSR-status completed two standardized executive function tasks, filled out questionnaires and underwent an affective picture paradigm, which previously had been shown to trigger MUS in high HSR (22, 58). Interestingly, besides low NA-low HSR and high NA-high HSR, we only found high NA-low HSR persons and no low NA-high HSR ones. Further research using a representative sample is needed to support an interpretation of this finding as evidence that trait NA is a necessary but not a sufficient condition to develop MUS. Consistent with associative learning studies showing that high NA are particularly vulnerable to develop MUS in laboratory manipulations [15–17], this observation calls for longitudinal studies tracing the developmental course of the relationship between trait NA and MUS. By comparing these three groups, we wanted to investigate whether brief inductions of state NA would elicit elevated symptom reporting, whether this was depending on trait NA or on HSR, and whether individual differences in executive function and in some self-reported characteristics were related to this effect.

The results on executive function clearly show that deficient response inhibition is related to the broad trait of NA in general and not to HSR. High trait NA persons showed impaired inhibition on the Parametric Go/No-go Task, were slower and made more mistakes in the Stroop Task, compared with low trait NA persons. The fact that the Stroop interference effect did not differ significantly between the NA groups is probably due to the fact that the interference effect consists of a subtraction (reaction time to card C minus reaction time to card B), and that the difference in reaction times between the NA groups was already significant for both cards. Nevertheless, the high trait NA persons did make more errors on the response inhibition Card C. It is important to note that, whereas executive function tests distinguished high from low trait NA, differences in alexithymia and absorption were related to differences in HSR rather than in trait NA.

The results obtained with the affective picture paradigm showed that all participants reported higher levels of state NA during the negative picture series than during the positive and neutral pictures, but differences in state NA between the three groups only appeared during negative picture viewing. However, only high habitual symptom reporters also reported substantially more bodily symptoms after viewing the negative pictures. Multilevel mediation/moderation analyses showed that state NA was a moderator, not a mediator, in the effect of HSR on symptom reporting. This study corroborates in an fMRI compatible design earlier results of our group [22, 58], showing that merely watching negative pictures in a neutral nonmedical environment (i.e. without inducing nor measuring somatic responses) is sufficient to induce the experience of symptoms in high habitual symptom reporters. So, it appears that these emotion induction effects on symptom reports cannot be explained by the broad NA trait only, but in contrast are specific for persons with high HSR (who are a subset of high trait NA persons). Because the effects on symptom reporting are not mediated but moderated by state NA, our data allude to the role of learned somatic schemata being evoked by affective cues, rather than by experiencing the negative affective state per se, which subsequently bias the experience of one’s bodily sensations [1]. In line with this, a recent study showed that both the valence and arousal characteristics of the cues contribute to the symptom reporting effect [58]. Because the affective picture paradigm replicates previous findings in a fMRI compatible format, two important advantages are illustrated: the paradigm robustly withstands major changes in parameters, and it shows that it can be used in future neuroimaging research to investigate in a standardized way the abnormalities in central processing of internal states that may underlie MUS.

The symptom induction effect (symptom difference between neutral and negative picture viewing) in the affective picture paradigm was not related to individual differences in executive function, but was positively associated with the tendency to become “absorbed” in one’s experiences and with the difficulty in identifying one’s feelings (F1 scale of TAS-20). The absorption findings are particularly intriguing, since they are exclusively elevated in high HSR persons and the items of the questionnaire do not imply a negative affective quality. Absorption has been linked to female gender, heightened creativity, hypnotisability, imagery-vividness, fantasy-proneness, and day-dreaming [49–51]. Artistic and aesthetic minded people seem to score higher on this questionnaire as well [52–54]. Individuals high in hypnotic susceptibility may be particularly sensitive to the automatic activation of cognitive and behavioral schemata by internal and external events [55], which could increase their vulnerability to developing somatoform symptoms [1]. According to Witthöft and colleagues [56], idiopathic environmental intolerance (IEI)—a condition marked by MUS, which patients attribute to various chemical odorous substances—was specifically related to elevated absorption scores, compared with other somatoform disorders and healthy controls. However, in the affective picture paradigm, the addition of absorption and alexithymia as covariates did not improve prediction of symptom reports. This finding suggests that, although absorption and alexithymia are associated with habitual symptom reporting, their role in explaining differential symptom reporting in the experimental symptom induction is limited. However, further research into the specific pathways in which absorption may contribute to high HSR is warranted.

An important limitation of this study is that we drew our participants from a homogeneous population of young, non-clinical students. Several findings (role of state NA, relation with absorption, loose relationship between somatic challenges and symptom reports) suggest that in the clinical MUS population the same mechanisms may apply in a magnified form as in the non-clinical group, a replication of the results in a clinical group is needed. Secondly, our study sample was confined to women. Given that women are more likely to suffer from MUS than men [2], this may be justifiable, but gender-related differences regarding the type of symptoms that relate most strongly to trait NA [8] call for a replication with men. Future studies should also clarify whether the present findings are generalizable to other populations (e.g., older people with varying socio-economic status and different MUS patient groups).

Our findings may have important clinical implications. In line with the idea behind interoceptive exposure in panic disorder patients [57] directing attention towards bodily sensations in an objectifying, neutral manner (as opposed to a negative affective way) may be particularly beneficial to inhibit the automatic activation of symptom schemata. Also, if scoring high on absorption makes people more vulnerable to the “nocebo”-effect, new studies should investigate whether they are also more susceptible for the “placebo”-effect, possibly providing interesting suggestions for clinical interventions.

In sum, our findings add to understanding the widely reported relationship between trait NA and elevated symptom reporting. First, our data suggest that trait NA is a vulnerability factor but not a sufficient condition to show MUS. Second, elevated symptom reporting can easily be elicited by presenting negative affective cues, but this effect is confined to high HSR. Third, two well-established indirect measures of prefrontal inhibitory control indicate that deficient inhibition is related to the broad trait of NA and but not to HSR. Fourth, characteristics such as alexithymia and absorption are elevated in high HSR individuals and correlate with the increase in symptom reports after negative picture viewing. Further research into pathways that may explain this association may prompt better understanding of medically unexplained symptoms.

## Supporting Information

S1 File This file contains the raw data of the affective picture paradigm. Data S1, Contains the raw data of the high NA-high HSR participants. Data S2, Contains the raw data of the high NA-low HSR participants. Data S3, Contains the raw data of the low NA-low HSR participants.
(ZIP)

S2 File This data set contains the data per participant for the Parametric Go No Go task, Stroop Color Word task, and trait questionnaires PANAS-trait (NA-scale), CSD, Toronto Alexithymia Scale (and subscales) and the Tellegen Absorption Scale. (STA)

S1 Text This text lists the pictures selected from the IAPS set (http:// csea.phhp.ufl.edu/media/iapsmessage.html). (DOCX)
