# Supplementary material for: Unraveling the Relationship between Trait Negative Affectivity and Habitual Symptom Reporting
Source: PLoS One. 2015 Jan 20;10(1):e0115748. doi: 10.1371/journal.pone.0115748 (PMC4300148; doi:10.1371/journal.pone.0115748)
Supplement: S1 Text — (DOCX) [file pone.0115748.s003.docx]

**Neutral Pictures:**

1121; 1560; 1670; 1675; 1850; 1942; 1947; 2025; 2038; 2102; 2104; 2190; 2191; 2200; 2206; 2210; 2214; 2235; 2272; 2305; 2357; 2372; 2381; 2383; 2385; 2393; 2396; 2397; 2435; 2445; 2480; 2485; 2487; 2495; 2499; 2514; 2518; 2575; 2579; 2580; 2593; 2594; 2597; 2850; 2870; 2880; 2980; 5395; 5455; 5471; 5520; 5740; 7002; 7004; 7036; 7037; 7041; 7130; 7140; 7205; 7217; 7491; 7493; 7495; 7496; 7504; 7506; 7546; 7550; 7640; 8211; 8311

**Positive Pictures:**

1340; 1463; 1603; 1620; 1710; 1731; 1750; 1920; 2058; 2165; 2209; 2216; 2299; 2304; 2311; 2340; 2341; 2345; 2360; 2387; 2388; 2395; 2398; 2501; 2530; 2550; 2598; 2660; 4532; 4574; 4610; 4614; 4622; 4626; 4640; 5001; 5010; 5201; 5260; 5480; 5551; 5621; 5623; 5700; 5760; 5811; 5831; 5833; 5836; 5849; 5994; 7230; 7280; 7282; 7325; 7340; 7502; 7580; 8162; 8170; 8185; 8210; 8370; 8380; 8420; 8461; 8470; 8496; 8497; 8499; 8502; 8540

**Negative Pictures:**

1114; 1200; 1302; 1932; 2095; 2120; 2683; 2691; 2692; 2694; 2703; 2751; 2799; 2800; 2811; 2900.1; 3500; 3530; 4621; 4635; 5971; 5973; 6020; 6190; 6212; 6241; 6242; 6250.1; 6312; 6313; 6315; 6370; 6550; 6560; 6571; 6800; 6821; 6838; 6840; 6940; 8485; 9001; 9006; 9041; 9050; 9140; 9181; 9220; 9230; 9270; 9340; 9342; 9404; 9409; 9410; 9417; 9419; 9421; 9423; 9424; 9425; 9426; 9429; 9440; 9470; 9520; 9561; 9600; 9622; 9800; 9900; 9911
